# Compound heterozygous *KCNV2* variants contribute to cone dystrophy with supernormal rod responses in a Chinese family

**DOI:** 10.1002/mgg3.1795

**Published:** 2021-09-18

**Authors:** Man Liu, Yingchuan Zhu, Lian Huang, Wenhao Jiang, Na Wu, Yue Song, Yilu Lu, Yongxin Ma

**Affiliations:** ^1^ Department of Medical Genetics State Key Laboratory of Biotherapy West China Hospital Sichuan University Chengdu China

**Keywords:** compound heterozygous mutations, cone dystrophy with supernormal rod response, exome sequence, *KCNV2*

## Abstract

**Background:**

Cone dystrophy with supernormal rod response (CDSRR) is an autosomal recessive retinal disorder characterized by myopia, dyschromatopsia, nyctalopia, photophobia, and nystagmus. CDSRR is caused by mutations in *KCNV2*, the gene encoding for an electrically silent Kv subunit (Kvs) named Kv8.2.

**Methods:**

A Chinese CDSRR family was recruited. Complete ophthalmology clinical examinations were performed to clarify the phenotype. Genetic examination was underwent using whole exome sequencing (WES). In addition, a candidate gene was validated by Sanger sequencing. Expression analysis in vitro including immunoblotting, quantitative real‐time PCR (qRT‐PCR), and co‐immunoprecipitation experiments was performed to investigate the pathogenic mechanism of the identified gene variants.

**Results:**

WES identified two *KCNV2* heterozygous mutations from the proband. Sanger sequencing validated that the patient's parents had, respectively, carried those two mutations. Further in vitro functional experiments indicated that the mutated alleles had led the Kv8.2 proteins to fail in expressing and interacting with the Kv2.1 protein, respectively.

**Conclusions:**

This study expanded the *KCNV2* mutation spectrum. It can also be deduced that CDSRR has a broad heterogeneity. It is further confirmed that the inability expression of Kv8.2 proteins and the failure of Kv8.2 proteins to interact with Kv2.1 may have accounted for the etiology of CDSRR based on previous studies and this study.

## Graphical abstract

Compound heterozygous variations (c.731G>C and c.280dup) were revealed in the KCNV2 gene of a male with CDSRR. Functional analysis had confirmed that c.280dup may led to non‐sensed mRNA decay (NMD), which in turn makes the protein unable to express. In addition, it is also approved that the c.731G>C mutation, in which the Kv2.1 protein failed to interact with Kv8.2 proteins. For the etiology of CDSRR, our study suggested that the inability expression of Kv8.2 proteins along with the failure of interactions between the Kv2.1 protein and the Kv8.2 proteins may account for the occurrence of the CDSRR.

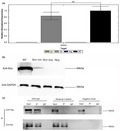

## 1 INTRODUCTION

Cone dystrophy with supernormal rod response (CDSRR; OMIM#610356) was first reported in two of four children in a Hispanic family (Gouras et al., 1983). Subsequent studies confirmed CDSRR as a retinal disorder inherited in an autosomal recessive manner with special electroretinogram (ERG) responses, which constitutes subnormal rod b‐wave in dim flash, reduced, and delayed cone responses, but a normal or increased rod amplitude at a high‐intensity stimuli (Wissinger et al., 2008). Moreover, due to structural retinal abnormalities and macular retinal pigment epithelial change or atrophy caused by CDSRR (Vincent et al., 2013), patients are typically affected by mild to moderate myopia, dyschromatopsia, nyctalopia, photophobia, and nystagmus before 12 years of their lives (range from 0 to 11 years, the mean age of onset was 3.9 years old; Georgiou et al., 2021; Lenis et al., 2013; Michaelides et al., 2005; Robson et al., 2010). In addition, broad heterogeneity in phenotypes has been confirmed. For instance, nyctalopia sometimes occurs, with older patients always presenting typical FAF images in central areas of RPE atrophy circled by a ring‐like high density (Robson et al., 2010). Maculopathy may worsen with age, but nystagmus frequently improves along with the growth of patients (Khan et al., 2012). Clinical details, pathognomonic electrophysiology findings, and molecular genetical tests are essential for diagnosing the disorder (Grigg et al., 2013). Consequently, pathognomonic electrophysiology findings, especially electroretinogram (ERG) examination, usually direct the molecular genetical tests.

Genetic studies have associated the abnormality on the mutations of gene *KCNV2* (OMIM* 607604) on chromosome 9p24.2, which contains 2 exons and encodes a voltage‐gated potassium channel protein consisting of 545 amino acids. The *KCNV2* gene is expressed in the inner segments of photoreceptors and encodes an electrically silent Kv subunit (Kvs) named Kv8.2 (used to be termed Kv11.1). Kv8.2 belongs to silent channel protein which cannot produce a permanent outward flow of potassium in dark conditions. However, when the Kv8.2 proteins co‐expresses with the protein of Kv2 subfamily to form a complex, it will influence channel properties to perform physiological functions in the form of a functional heteromeric channel. The main structure of Kv8.2 proteins is composed of four α‐subunits surrounding a central aqueous pore. For each α‐subunit, it mainly contains the following domains: N‐terminal A and B (NAB) boxes, six transmembrane (TM) domains (S1–S6), and a pore loop (P) between S5 and S6. Among these functional domains, S4 functions as an active voltage sensor, while P contains a signature GYG sequence that plays a role in specifying the channel's potassium selectivity (Bocksteins & Snyders, 2012).

Here, we report a Chinese CDSRR patient with compound heterozygous variations in *KCNV2* gene (c. 280dup and c.731G>C). In addition to phenotypes, we have carried out some functional explorations on the exact pathogenic mechanism of these variants and provided some references for study of the etiology.

## 2 MATERIALS AND METHODS

### 2.1 Subjects and clinical examinations

The proband recruited was a 29‐year‐old male from a non‐consanguineous family in Sichuan Province, of Han Chinese origin. His medical records were reviewed, and the following information from his ophthalmological examination at the current age of 29 years was obtained: best‐corrected visual acuity (BCVA), color vision test, scanning laser ophthalmoscopy (SLO; Optos plc, Dunfermline), fundus autofluorescence (FAF), spectral domain optical coherence tomography (SD‐OCT; Heidelberg Engineering), full‐field electroretinogram (FFERG), and visual field testing in automated perimetry (Octopus, Haag Streit international, Switzerland). FFERG was performed according to the minimum standard protocols of ISCEV (McCulloch et al., 2015). The ERGs examination included the following: (a) dark‐adapted dim flash 0.01 cd•s•m^−2^ ERG (DA 0.01), (b) dark‐adapted bright flash 3.0 cd•s•m^−2^ ERG (DA 3.0), (c) dark‐adapted oscillatory potential ERG, (d) light‐adapted 3.0 cd•s•m^−2^ at 2 Hz ERG (LA 3.0), and (e) light‐adapted 3.0 cd•s•m^−2^ 30 Hz flicker ERG (LA 3.0 30 Hz). Then, comparing the ERG data of patient with healthy control group (ages from 18 to 70 years, *n* = 141).

### 2.2 Mutation screening

Collected peripheral whole blood samples of all pedigree members with EDTA anticoagulation tubes, and then extracted gDNA for exome sequencing. The gDNA sample was sheared with ultrasound. The sheared gDNA was hybridized with Roche's NimbleGen 2.0 probe sequence capture array (http://www.nimblegen.com/products/seqcap/ez/v2/index.html) to enrich exon DNA (Joy Orient). First, the enrichment of the library was detected by qPCR, the size and concentration of the library were detected using the Agilent 2100 Bio‐analyzer. Two parallel reactions were performed on each sample and Illumina HiSeq 2500 platform was used to sequence the samples. The original image file was processed by Bcl2Fastq (Illumina) for basic calling and generating original data. BWA was used to compare the sequencing sequence with the NCBI human reference genome (hg19). Single nucleotide polymorphism (SNP) and indel analysis of the sequence were performed with Samtools and Pindel. The data analysis method was as follows: referring to the NCBI database, synonymous variations and SNPs, data with MAF ≥ 5% were deleted; non‐synonymous variations were filtered using SIFT software; the function of the mutant gene and its relationship with the disease were analyzed. Sanger sequencing was employed to further verify the variant sites of suspected pathogenic genes in the pedigree.

### 2.3 Mutated *KCNV2* construction

To further explore the effects of *KCNV2* variations in vitro, the *KCNV2* (NM_133497.4) referred to human wild‐type and mutant coding sequences were constructed into the pcDNA3.1(+) expression vectors with an N‐terminal MYC‐tag. Moreover, the human wild‐type sequence of *KCNB1* (NM_004975.4) was constructed into the pENTER vector based on the commercial plasmids, which contains a C‐terminal FLAG‐tag. Finally, sequenced all constructs before the experiment.

### 2.4 Cell culture and transfection

In this study, HEK‐293T cells were cultured as the cell line expressing plasmids. For transfection, cells were cultured on 6‐well plate and transfected at high density (~70%) using 2.5 µl transfection reagent (Lipofectamine 3000 Reagent, Life Technology) and 2.5 µl total plasmid.

### 2.5 Quantitative real‐time PCR

After transfecting for 48 hr, the whole RNAs were extracted from experimental samples (Mut‐mis and Mut‐dup) and control samples (WT and Neg), and quantitative real‐time PCR (qRT‐PCR) analysis was performed to examine the mRNAs level. cDNAs were synthesized in 20 µl reaction volume. The primer sequences are listed in Table 1. The exact reaction system and conditions for qRT‐PCR refer to Tables 2, 3 and 2, 3.

**TABLE 1:** qRT‐PCR primer sequences

miRNA	Primer	Sequence (5′−3′)
*KCNV2*	*KCNV2*‐F	CCTGGAACACGACGGAGA
	*KCNV2*‐R	TGCCAGGTCGTCCTTCCA
*GAPDH*	*GAPDH*‐F	AAGGTGAAGGTCGGAGTCAA
	*GAPDH*‐R	AATGAAGGGGTCATTGATGG

**TABLE 2:** qRT‐PCR reaction system

Reagent	Volume of use
2xchamQ Universal SYBR qPCR Master Mix (Vazyme, China)	10.0 µl
Primer F (10 µM)	0.5 µl
Primer R (10 µM)	0.5 µl
Template DNA/cDNA	1 µl
Distilled deionized water (ddH_2_O)	Up to 20 µl

**TABLE 3:** qRT‐PCR reaction conditions

Stage	Temperature	Reaction time
Initial denaturation	95℃	3 min
Cyclic reaction (35 cycles)	95℃	10 sec
	60℃	10 sec
Melting curve analysis	95℃	15 sec
	60℃	60 sec
	95℃	15 sec

### 2.6 Immunoblotting

Immunoblotting (IB) experiment was performed with the anti‐MYC (Proteintech) antibodies to analyze each protein lysate. Anti‐GAPDH was used as an internal control antibody. The specific experimental protocol of IB experiment referred to the paper published by Zhu et al. (2019).

### 2.7 Co‐immunoprecipitation

The FLAG‐tagged Kv2.1 was co‐transfected with MYC‐tagged wild‐type Kv8.2, missense‐mutated Kv8.2, dup‐mutated Kv8.2, and empty vector pcDNA3.1(+) into the HEK‐293 cells in a 3:1 ratio (Smith et al., 2012) using a jetPRIME transfection kit (Lipofectamine 3000 Reagent, Life Technology), respectively. To probe the interactions of the mutated monomers of the *KCNV2* proteins with Kv2.1, co‐immunoprecipitation (co‐IP) analyses were performed on the extracted protein lysates. After a part of the protein lysates from the samples used for positive control (input) were extracted, the remaining proteins were divided into the experimental group (IP) and the negative control (IgG) group. Then, 1 µg of anti‐MYC (Proteintech) and normal IgG was added (Beyotime) into the IP group and IgG group, respectively. After that, 2 µg of protein beads (Beyotime) was added into each tube of sample, and placed the mixed solution in 4℃ environment and incubated overnight. The next day, 40 µl of protein beads was added to each mixture and incubated at 4℃ for 3 hr. Then, the beads were washed for three times with a pre‐cooled PBS solution, and finally the protein denaturation was used for IB experiment with anti‐FLAG and anti‐MYC antibodies to take a further exploration.

## 3 RESULTS

### 3.1 Subjects and clinical findings

**FIGURE 1:**
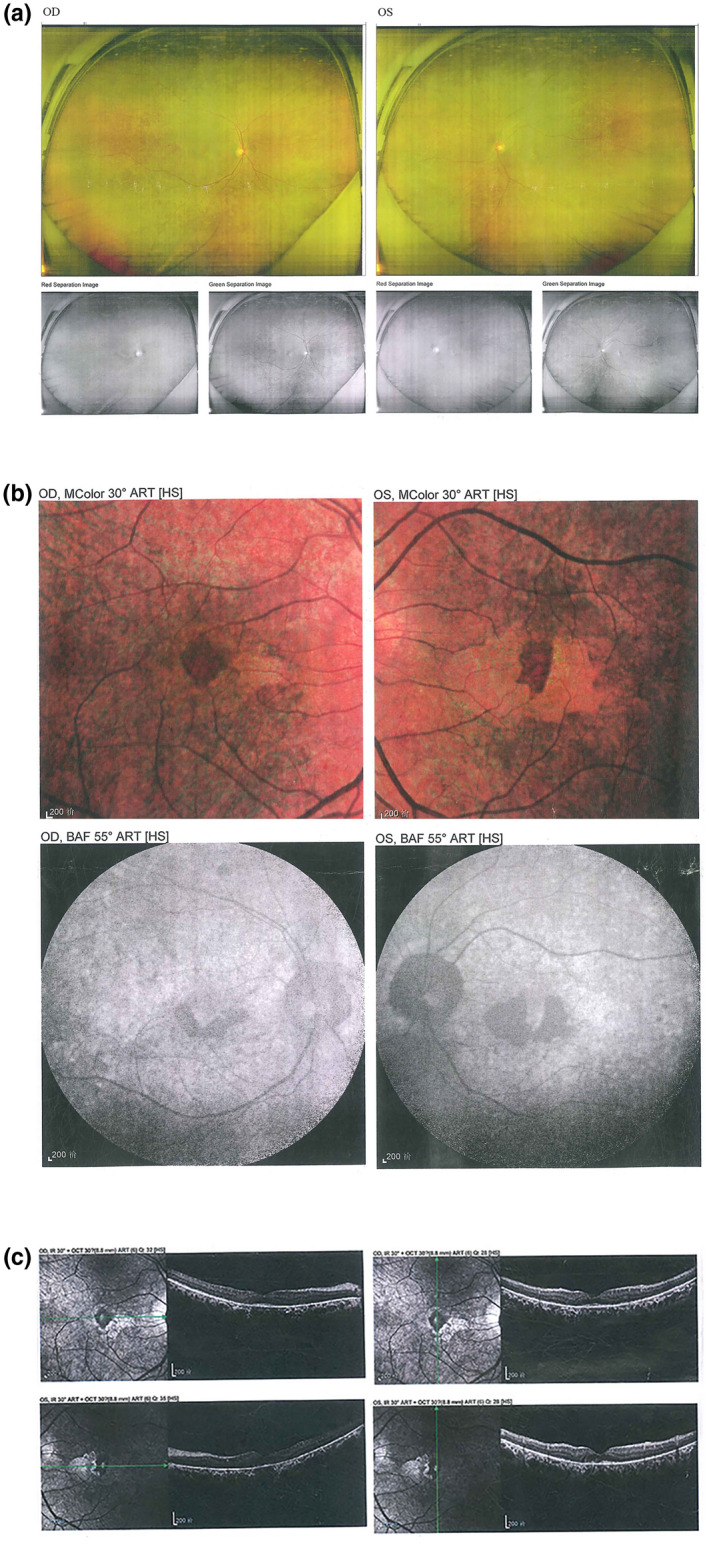
Scanning laser ophthalmoscope (SLO), fundus autofluorescence (FAF), and optical coherence tomography (OCT) scans in the proband (II 1). (a) Scanning laser ophthalmoscopy (SLO) images showed slightly thin blood vessels in the fundus of both eyes. (b) Fundus autofluorescence (FAF) examination showed irregular autofluorescence in the macular area of both eyes. (c) Spectral domain optical coherence tomography (SD‐OCT) indicated the photoreceptor layer become thin and the ellipsoid zone was discontinuous in both eyes. OD right eye, OS left eye

**FIGURE 2:**
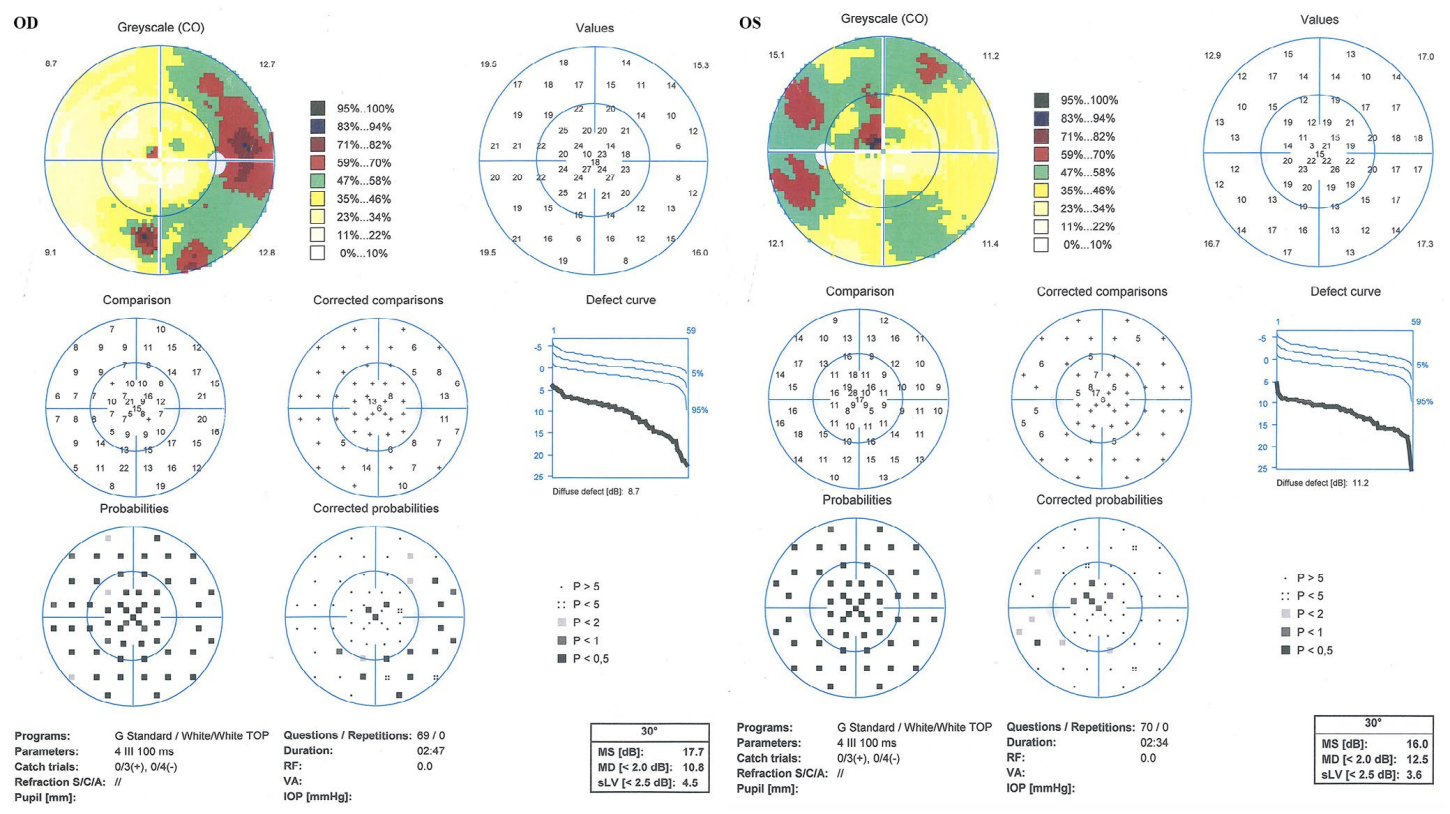
Goldmann visual field testing characteristics of the proband (II 1). Goldmann visual field testing showed there is partial defect of the central visual field in both eyes. OD right eye, OS left eye

**FIGURE 3:**
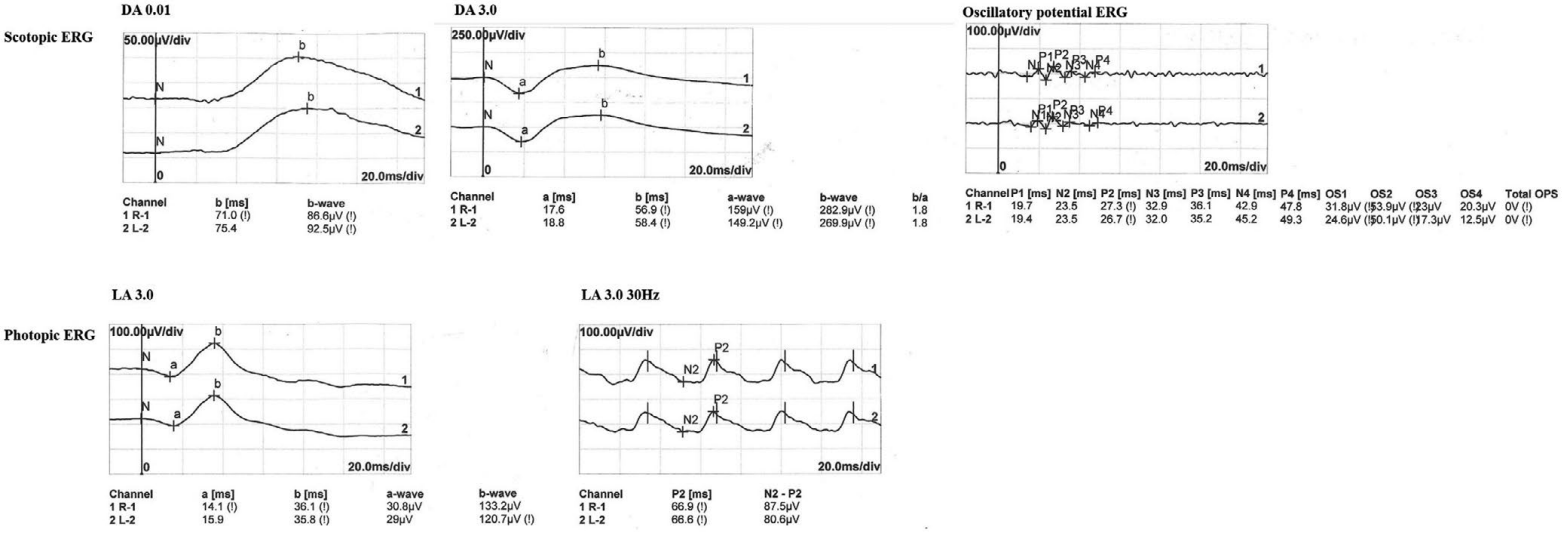
Full‐Field electroretinogram of the proband (II 1). Full‐field electroretinogram (FFERG) showed decreased amplitudes in b‐waves for DA 0.01, delayed peak time but a normal amplitude in b‐wave and diminished a‐wave for DA 3.0, decreased amplitudes in a‐wave and delayed peak time in b‐wave for LA 3.0, delayed implicit time for LA 3.0 30 Hz. DA 0.01 dark‐adapted dim flash 0.01 cd•s•m^−2^ ERG, DA 3.0 dark‐adapted bright flash 3.0 cd•s•m^−2^ ERG, LA 3.0 light‐adapted 3.0 cd•s•m^−2^ at 2 Hz ERG, and LA 3.0 30 Hz light‐adapted 3.0 cd•s•m^−2^ 30 Hz flicker ERG

**FIGURE 4:**
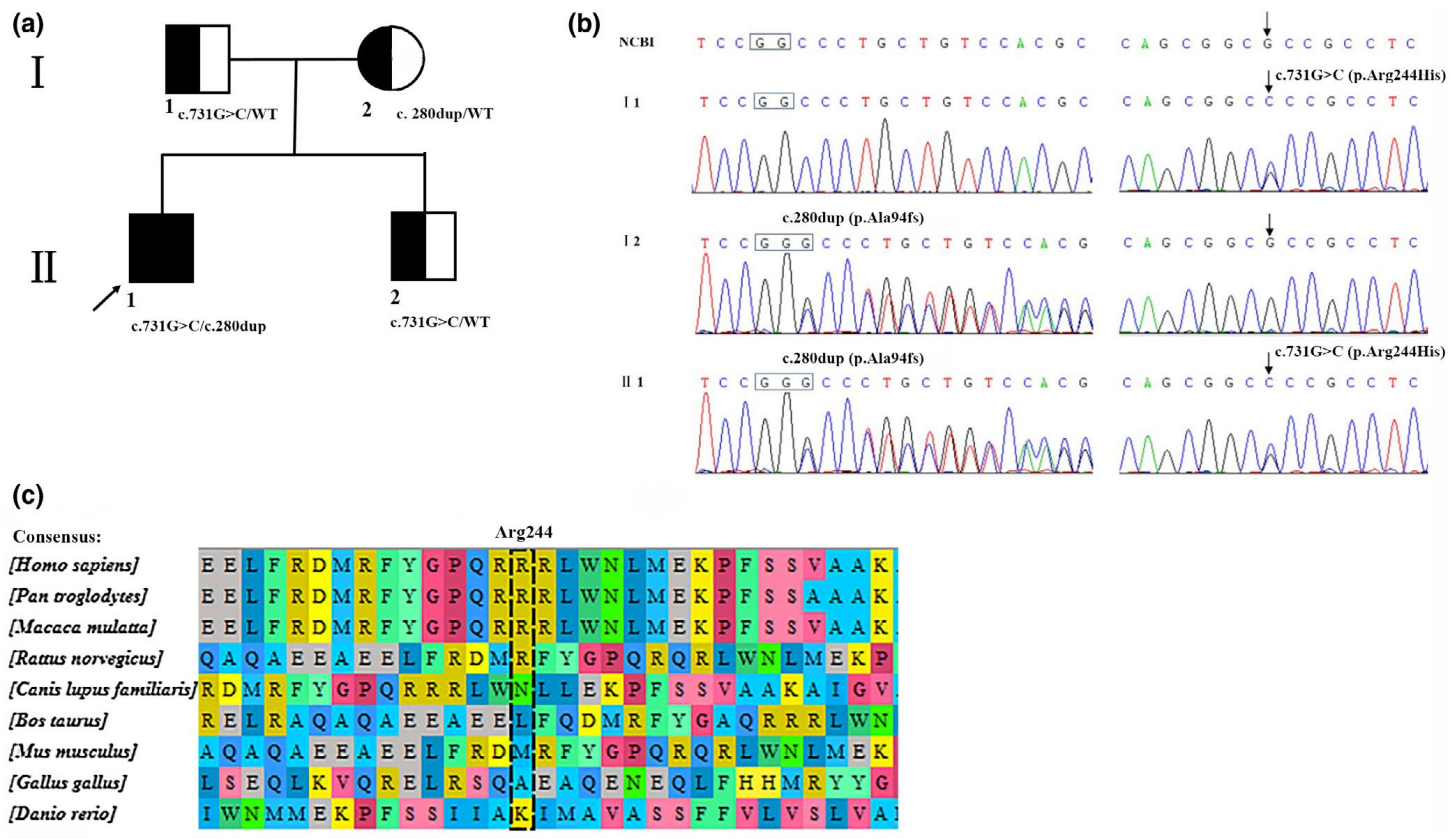
Family pedigree of the CDSRR proband and the mutation transmission of the *KCNV2* (NM_133497.4) gene. A Family pedigree of the proband. B Sequence analysis shows that the father (I 1) was heterozygous at c.731G>C in *KCNV2* gene, the mother (I 2) was heterozygous at c.280dup in *KCNV2* gene, and the proband (II 1) was heterozygous at both sites. C Comparison of the amino acid sequences near the Arg244 of *KCNV2* from different organisms. The box marked with black dotted line marked the site of the mutated amino acids

The family's pedigree is illustrated in Figure 4a, in which one member suffered from CDSRR. The proband complained of decreased visual acuity on both eyes, photophobia and night blindness, and had been suffering from nystagmus since 4 years old. Upon further examination during his early childhood, a red–green axis of dyschromatopsia was observed. Until the patient's 29 years old, the BCVAs were 0.83 logMAR (OD) and 0.88 logMAR (OS). Slightly thin blood vessels were identified from the SLO (Figure 1a). The FAF demonstrated irregular autofluorescence in the macular area of both eyes (Figure 1b). The SD‐OCT findings presented that the photoreceptor layers of both eyes became thin and the ellipsoid zone was discontinuous (Figure 1c). Automated visual field testing revealed partial defects in the central visual field of the patient's eyes (Figure 2). The proband's electrophysiological details are summarized in Figure 3 and Table 4. The amplitude of DA0.01 ERG b‐wave was decreased. For the DA3.0 ERG, a‐wave in both eyes demonstrated a normal peak time but a subnormal amplitude, while the b‐wave peak time was delayed and amplitude was normal. The LA 3.0 ERG a‐wave was subnormal and b‐wave demonstrated delayed peak time. The LA 3.0 30 Hz ERG had delayed peak time and decreased amplitude.

**TABLE 4:** The amplitudes and peak times of the full‐field electroretinography components in control group compared with the patient.

	DA0.01 ERG		DA3.0 ERG				Oscillatory potential ERG		LA 3.0 ERG				LA 3.0 30 Hz ERG	
	b‐wave		a‐wave		b‐wave		OS1	OS2	a‐wave		b‐wave		b‐wave	
	Peak time (ms)	Amplitude (µV)	Peak time (ms)	Amplitude (µV)	Peak time (ms)	Amplitude (µV)	Amplitude (µV)	Amplitude (µV)	Peak time (ms)	Amplitude (µV)	Peak time (ms)	Amplitude (µV)	Peak time (ms)	Amplitude (µV)
Control	[66.92, 80.07]	[112.28, 282.58]	[16.4,19.69]	[222.66, 349.95]	[36.7,42.29]	[271.43, 573.31]	[25.6,69.9]	[49.41, 132,38]	[12.9,15.6]	[35.35,67.79]	[29.1,31.69]	[78.12, 175.57]	[59.92,63.69]	[95.1, 197.2]
Patient														
R	71.0	86.6	17.6	159	56.9	282.9	31.8	53.9	14.1	30.8	36.1	133.2	66.9	87.5
L	75.4	92.5	18.8	149.2	58.4	269.9	24.6	50.1	15.9	29	35.8	120.7	66.6	80.6

Note

The ERGs values from normal control are shown for comparison.

Abbreviations: DA, dark‐adapted; ERG, electroretinogram; L, left eye; LA, light‐adapted; R, right eye.

### 3.2 Whole exome sequencing

The *KCNV2* mutations were found in both the proband and his families. First, compound heterozygous variations at sites c.280dup (Genome position: chr9: 2718071_2718018, rs1221249050) and c.731G>C (Genome position: chr9:2718470, rs1023306854) of the *KCNV2* gene were detected in the proband (Figure 4a), resulting in a frameshift mutation of p. Ala94fs and missense mutation of p. Arg244Pro. Further sequencing verified that the proband's mother and father carried the c.280dup and c.731G>C of the *KCNV2* gene, respectively (Figure 4b). The exact pathogenicity and clinical significance of these two mutations of the *KCNV2* gene had not been reported previously.

### 3.3 Genetic variation analysis

Regarding the missense mutation c.731G>C (p. Arg244Pro) of the *KCNV2* gene, the amino acid residue Arg244 was moderately conserved in *KCNV2* (Figure 4c). Additionally, PolyPhen‐2 (score: 0.876) and deleterious through PROVEAN (score: −3.503) showed that c.731G>C was a possibly damaging mutation. Alongside the proband's clinical manifestations and based on the ACMG standards and guidelines (Richards et al., 2015), two pathogenicity moderate criteria (PM1 and PM2) and two pathogenicity supporting criteria (PP1 and PP4) were identified for variant c.731G>C (p. Arg244Pro; Evidence of pathogenicity: (a) PM1: Moderate: the variant located in a mutational hot spot; (b) PM2: Moderate: absent from controls in Exome Sequencing Project, 1000 Genomes Project, or Exome Aggregation Consortium; (c) PP1: Supporting: mutations and diseases co‐segregate in the family; (4) PP4: Supporting: patient's phenotype is highly specific with the CDSRR with a single genetic etiology). Therefore, according to this scoring rules, variant c.731G>C (p. Arg244Pro) should be classified as “Likely pathogenic.” With regard to another variation c.280dup (p. Ala94fs), one pathogenicity very strong criterion (PVS1), two pathogenicity moderate criteria (PM2 and PM4), and two pathogenicity supporting criteria (PP1 and PP4) were present (Evidence of pathogenicity: (a) PVS1: Very strong: the loss of function (LOF) of *KCNV2* gene is a known mechanism of CDSRR. In this study, the *KCNV2* gene had a null variation; (b) PM2: Moderate: absent from controls in Exome Sequencing Project, 1000 Genomes Project, or Exome Aggregation Consortium; (c) PM4: Moderate: protein length changed as a result of frameshift mutation; (d) PP1: Supporting: mutations and diseases co‐segregate in the family; (e) PP4: Supporting: patient's phenotype is highly specific with the CDSRR with a single genetic etiology). Thus, variant c.280dup (p. Ala94fs) should be classified as “pathogenic.”

### 3.4 In vitro analysis of *KCNV2* mutations

**FIGURE 5:**
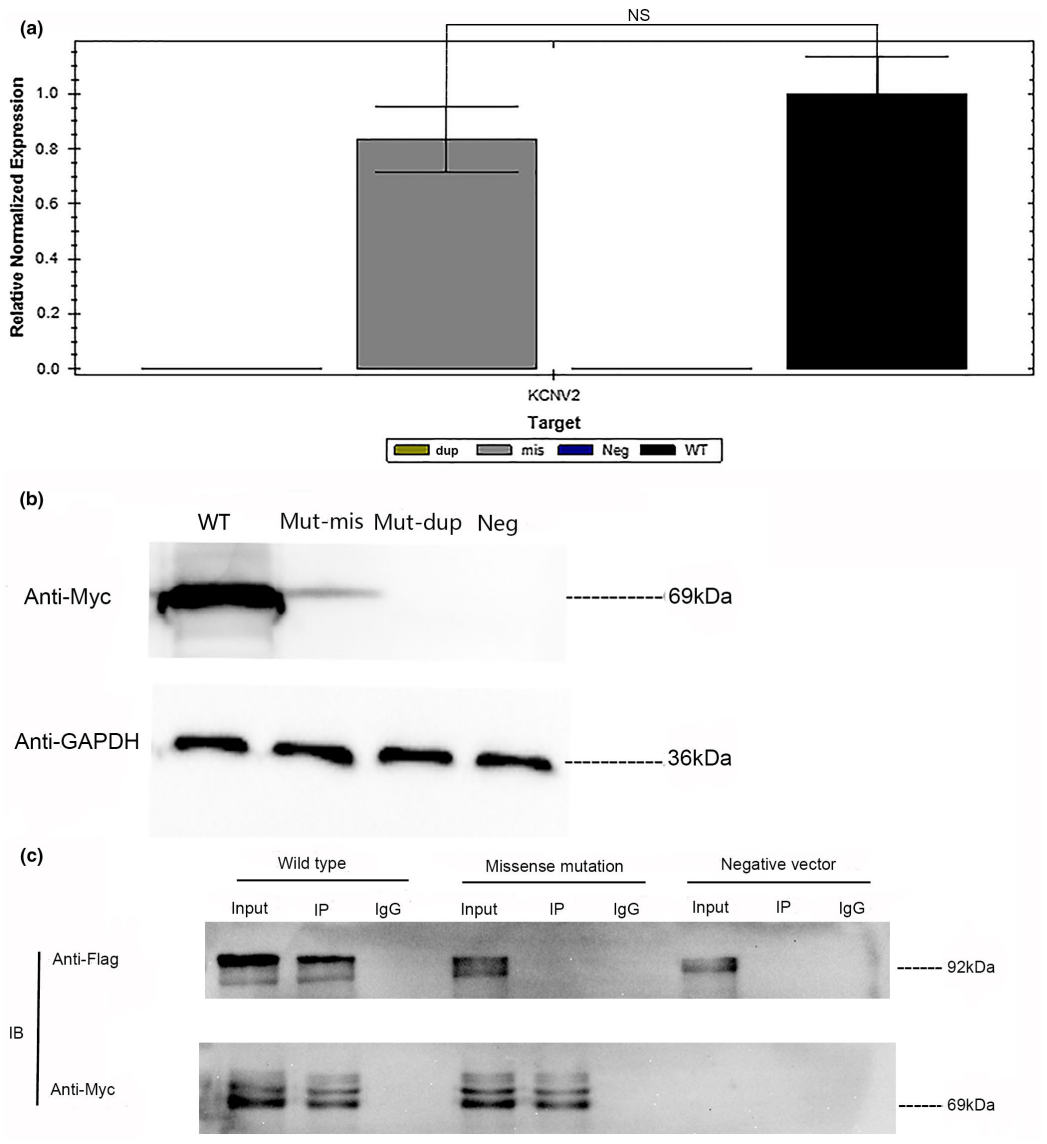
In vitro analysis of the *KCNV2* (NM_133497.4) gene mutations. A The relative mRNA expression levels of wild‐type *KCNV2* and mutant Arg244Pro and Ala94fs in 293T cell was evaluated using quantitative real‐time PCR analysis. WT, mis, dup, and Neg refers to the relative mRNA expression of wild type, mutant Arg244Pro *KCNV2*, Ala94fs *KCNV2*, and empty vector, respectively. Data are shown as mean ± standard error of the mean (NS, no significance, *n* = 3). B Proteins expression of the wild type and two mutant *KCNV2* were examined by the immunoblot analysis with anti‐Myc antibodies. GAPDH was used as an internal control. The result indicates the protein products of wild‐type *KCNV2* (WT), *KCNV2* Arg244Pro (Mut‐mis), *KCNV2* Ala94fs (Mut‐dup), and negative vector (Neg). C Interactions of the wild‐type *KCNV2*‐N‐Myc and Arg244Pro‐N‐Myc proteins with Kv2.1‐C‐Flag protein were analyzed by co‐IP analysis with the anti‐Myc antibody and IB analysis with an anti‐Flag antibody. The result shows that the protein Arg244Pro‐N‐Myc cannot be immune‐precipitated by Kv2.1‐C‐Flag

The qRT‐PCR analysis showed that Ala94fs had a very low mRNA expression and mRNA expression of Arg244Pro was decreased non‐significantly compared with the WT *KCNV2* (Figure 5a). The IB analysis further corroborated the results of qRT‐PCR, which demonstrated that Ala94fs protein was fail to express, and Arg244Pro was less expressed compared with the WT *KCNV2* protein (Figure 5b). Additionally, the subsequent co‐IP analysis demonstrated that the WT *KCNV2* could normally interact with the Kv2.1‐c‐Flag protein, while Arg244His proteins and the negative vector had all failed to interplay with the Kv2.1‐c‐Flag protein (Figure 5c). These results signified that the Ala94fs mutation led to a failure expression of *KCNV2* because of mRNAs degradation, and Arg244Pro mutation had indeed prevented the Kv8.2 proteins and Kv2.1‐c‐Flag protein from interacting.

## 4 DISCUSSION

According to Robson (Robson et al., 2010) and Khan (Khan et al., 2012), there was variability in the phenotypes of CDSRR patients in that nystagmus and nyctalopia were common symptoms in youth patients, and nystagmus symptom could even show spontaneous resolutions with increasing age. In this study, the patient suffered from a decreased central vision since early childhood, along with clinical presentations of nystagmus, photophobia, dyschromatopsia, and night blindness. These symptoms were all in accordance with the presentations of CDSRR (Michaelides et al., 2005; Robson et al., 2010; Tanna et al., 2017; Wissinger et al., 2008, 2011). However, there were certain differences. For instance, this case had suffered from nystagmus and nyctalopia since early childhood, but he complained that the nystagmus without any reduce with age. We suspected that this may be for the fact that the patient is still relatively young, and as his age increases, nystagmus may improve. Overall, these findings might further validate that CDSRR had a large heterogeneity in the phenotype (Vincent et al., 2013).

The characteristic changes for of FFERG in CDSRR patients included: decreased amplitudes and delayed peak times for the rod‐mediated dim flash (DA0.01 ERG), normal or supernormal b‐waves with or without broadened a‐waves for the DA3.0 ERG or DA10.0 ERG, and diminished cone responses in LA 3.0 ERG and LA 3.0 30 Hz flickers ERG (Ben Salah et al., 2008; Guimaraes et al., 2020; Robson et al., 2010; Sergouniotis et al., 2012; Wissinger et al., 2008). Khan et al. (2012) and Zelinger et al. (2013) determined that some of the CDSRR patients might have normal instead of supernormal amplitude rod response in a dark‐adapted environment. Moreover, because of broad genetic heterogeneities, many patients with the same genetic variation may have different phenotypes and numbers of cases showed normal rather than supernormal b‐wave amplitude with high‐intensity stimulus, it is common that many cases had been misdiagnosed as cone dystrophy and some other retinal disorders. Based on those findings, some researchers pointed out that the term “supernormal rod ERG” may be misleading. Regarding the patient in this case, we initially doubted that he was a cone‐rod dystrophy (CORD) patient rather than CDSRR for the reason that there was no characteristic supernormal rod response. As for this patient, the DA0.01 FFERG manifested diminished amplitude b‐waves, the DA3.0 ERG showed reduced amplitude a‐waves, a normal amplitude but delayed b‐wave. Additionally, LA 3.0 ERG showed a diminished amplitude a‐waves and a delayed b‐waves. While the LA 3.0 30 Hz ERG indicated that the peak time was prolonged and the amplitude was reduced. The ERG results revealed that, the patient's cone and rod cells have varying degrees of damage, the abilities of receiving and conducting signals by cone and rod cells were weakened. In addition, the strength of the bipolar cell layer (Müller cells) to receive or conduct signals was also reduced. This series of atypical changes instruct us to advise that patient undergo genetic testing to further clarify the diagnosis. Finally, we combined the clinical phenotype and the compound heterozygous mutation of the *KCNV2* gene suggested by the genetic testing, we diagnosed the patient as a CDSRR.

To date, there are 92 published *KCNV2* mutations, of which 75% (69/92) of the variations have been linked to CDSRR and 13.0% (12/92) have been associated with cone dystrophy (COD) or cone‐rod dystrophy (CORD; available at: http://www.hgmd.cf.ac.uk/ac/index.php). Some of these studies have determined that most of the amino acid changes occur in the P‐loop and N‐terminal segment of the Kv8.2 proteins. The N‐terminal includes NAB boxes, which also indicates the significance of these regions to the Kv8.2 function. Genetic testing is needed to further clarify the etiology and predict the progress of the disease (Huang et al., 2016). In this study, it was suspected that the patient's CDSRR was due to *KCNV2* variations following second‐generation sequencing, and that the family's co‐segregation was realized through Sanger sequencing. After further analysis, the identified variation p. Ala94fs in the study sites before the NAB boxes with the addition of 279 novel amino acids in the protein. The other mutations (p. Arg244Pro) transformed a moderately conserved amino acid between the first transmembrane segment and the NAB boxes. Both variations, particularly p. Ala94fs, might have large‐scale impacts on the formation of functional domains for Kv8.2 proteins. To provide a much higher level of confidence, additional validation by functional assays in vitro was first carried out in this study. Through IB experiment, we observed that the Arg244Pro led to a reduced expression of Kv8.2 proteins and the Ala94fs caused the Kv8.2 proteins without normal expression. Using qRT‐PCR and co‐IP experiments, we observed that Ala94fs variation showed a sharp decline in mRNAs levels, which was coincided with the results of IB. We suspected that it was the nonsense‐mediated mRNA decay (NMD) that induced the zero mRNA expression of Ala94fs. While the mutation Arg244Pro resulted an impaired interaction between Kv8.2 and Kv2.1 proteins. The data convincingly validated the genetic etiology of the case, and provided further investigation for the pathogenic mechanism of the two *KCNV2* variations, which might also prove that the N‐terminal regions of Kv8.2 play a significant role in the function of protein.

Some studies have presented that mutations at the N‐terminal of Kv8.2 proteins may prevent the formation of Kv channels or lead to the formation of a pure Kv2.1 homologous channel. Due to the lack of regulation by Kv8.2 proteins, the channel formed by pure Kv2.1 protein may result an abnormality in photoreceptor cells and lead to an abnormal electroretinogram (Gayet‐Primo et al., 2018). Therefore, variations in the *KCNV2* gene may cause its protein to fail to bind to the Kv2.1 protein, which may explain the pathogenesis of CDSRR. Although p. Arg244Pro and p. Ala94fs variations have been noted in ClinVar database previously, the compound heterozygous variation of the two mutations has not been reported yet. In addition, there are no in vitro functional experiments to verify the exact influences of the two mutations, and the pathogenic mechanism of these two mutations is still unproven. In this study, we reported the combination of p. Arg244Pro and p. Ala94fs for the first time, and carried out functional exploration of the two mutations in vitro tests. Eventually, we suspected that the rare compound heterozygous loss‐of‐function mutants in *KCNV2* gene lead to the occurrence of the CDSRR which inherited in an autosomal recessive manner, the proband's phenotype combined our experiments had manifested a failure *KCNV2* expression and a functional absence between the Kv8.2 proteins and the Kv2.1 protein.

It should be pointed out that our research has the following shortcomings: First, for the FFERG examination, the patient did not perform the DA10.0 ERG which can further diagnose the rod response on the basis of DA3.0 ERG. Second, because the proband had onset at early childhood, which is a long time away from the current year, and we cannot verify the exact onset time of each clinical symptom. For example, we have no way of knowing the BCVA of the patient at the early stage of onset, moreover, the patient complained that there was no improvement of nystagmus since he suffered from CDSRR. In view of above problems, we cannot directly judge whether there is a progressive change for CDSRR though numbers of studies showed that CDSRR is a progressive disorder. And with the patient's age growing, whether the symptom of nystagmus will improve, we need to have a further follow‐up and study.

To conclude, compound heterozygous variations (c.731G>C and c.280dup) were revealed in the *KCNV2* gene of a male with CDSRR, which presented nystagmus, photophobia, dyschromatopsia, and night blindness at an early age, but had uncharacteristic FFERG changes compared to generalized CDSRR patients. Functional analysis had confirmed that c.280dup may led to non‐sensed mRNA decay (NMD), which in turn makes the protein unable to express. In addition, it is also approved that the c.731G>C mutation, in which the Kv2.1 protein failed to interact with Kv8.2 proteins. Thus, it was proposed that the variations rs1221249050 and rs1023306854 in *KCNV2* gene might result in loss‐of‐function mutations. It is further identified that CDSRR indeed has a broad heterogeneity and the supernormal rod responses may not exist in all the CDSRR patients, phenotypes combined with genetic testing are essential for diagnosis of this disorder. For the etiology of CDSRR, our study suggested that the inability expression of Kv8.2 proteins along with the failure of interactions between the Kv2.1 protein and the Kv8.2 proteins may account for the occurrence of the CDSRR.

## CONFLICT OF INTEREST

All authors certify that they have no affiliations with or involvement in any organization or entity with any financial interest (such as honoraria; educational grants; participation in speakers’ bureaus; membership, employment, consultancies, stock ownership, or other equity interest; and expert testimony or patent licensing arrangements), or non‐financial interest (such as personal or professional relationships, affiliations, knowledge, or beliefs) in the subject matter or materials discussed in this manuscript.

## ETHICAL COMPLIANCE

This study was approved by the Ethics Committee on Biomedical Research, West China Hospital of Sichuan University (No. 2021‐15). All of the participants signed informed consent before the study. All procedures performed in studies involving human participants were in accordance with the Declaration of Helsinki.

## AUTHOR CONTRIBUTIONS

Overall design and drafting the manuscript: Man Liu; investigate the family history and collect the clinical data of the patient: Yingchuan Zhu, Lian Huang, and Wenhao Jiang; experimental data analysis: Na Wu, Yue Song, and Yilu Lu; corresponding author: Yongxin Ma. All authors read and approved the final manuscript.

## Data Availability

The data that support the findings of this study are available from the corresponding author upon reasonable request.
